# Co-Designing Aliviado Caregiving: An AI-Powered Mobile App For Supporting Care Partners in Managing BPSD

**DOI:** 10.1093/geroni/igaf122.2518

**Published:** 2025-12-31

**Authors:** Moroni Fernandez Cajavilca, Shih-Yin Lin, Benedict Guzman, Kimberly Cheng, Denise Lawson, Narges Razavian, Abraham Brody

**Affiliations:** University of Utah, Salt Lake City, Utah, United States; New York University Rory Meyers College of Nursing, New York, New York, United States; New York University Langone Health, New York, New York, United States; New York University, New York, New York, United States; Pop Services, White Plains, New York, United States; New York University, New York, New York, United States; New York University Rory Meyers College of Nursing, New York, New York, United States

## Abstract

Care partners (CP) of persons living with dementia (PLWD) often face complex, unpaid caregiving responsibilities, particularly in managing behavioral and psychological symptoms of dementia (BPSD) such as agitation. CPs lack day-to-day access to evidence-based non-pharmacological interventions (e.g., music therapy) and decision support for daily BPSD management. Mobile health interventions offer a promising solution to aid CPs in managing BPSD. This presentation details the development of a precision heuristic machine learning algorithm and human-centered design of Aliviado Caregiving, a mobile health application designed to support CPs’ self-management and prioritization of BPSD. We used the National Alzheimer’s Coordinating Center Uniform Data Set to create an XGBoost model, training it on 2360 patients (80% of the final cohort) and testing it on 590 patients (20%). The model achieved an area under the curve of .829 and an average precision-recall curve of .905. We then embedded the algorithm into the Aliviado Caregiving app and established a community advisory board of diverse CPs of PLWD (N = 8). The board members participated in two focus groups and eight individual interviews to co-design and iteratively refine the Aliviado Caregiving application. We conducted a reflexive thematic analysis on open-ended responses collected from all focus groups and interviews. We identified four qualitative themes: 1) User-Interface Design Preferences and Suggestions, 2) User-Friendly Language, 3) User-Concern Prioritization, and 4) Future Application Features. Applying co-design principles and partnering with diverse CPs improved our ability to enhance the acceptability and usability of Aliviado Caregiving for future testing in a pilot project.

